# The double burden of malnutrition in India: Trends and inequalities (2006–2016)

**DOI:** 10.1371/journal.pone.0247856

**Published:** 2021-02-25

**Authors:** Phuong Hong Nguyen, Samuel Scott, Derek Headey, Nishmeet Singh, Lan Mai Tran, Purnima Menon, Marie T. Ruel

**Affiliations:** 1 Poverty, Health and Nutrition Division, International Food Policy Research Institute, Washington, DC, United States of America; 2 FHI360, Hanoi, Vietnam; University of Western Australia, AUSTRALIA

## Abstract

Rapid urban expansion has important health implications. This study examines trends and inequalities in undernutrition and overnutrition by gender, residence (rural, urban slum, urban non-slum), and wealth among children and adults in India. We used National Family Health Survey data from 2006 and 2016 (n = 311,182 children 0-5y and 972,192 adults 15-54y in total). We calculated differences, slope index of inequality (SII) and concentration index to examine changes over time and inequalities in outcomes by gender, residence, and wealth quintile. Between 2006 and 2016, child stunting prevalence dropped from 48% to 38%, with no gender differences in trends, whereas child overweight/obesity remained at ~7–8%. In both years, stunting prevalence was higher in rural and urban slum households compared to urban non-slum households. Within-residence, wealth inequalities were large for stunting (SII: -33 to -19 percentage points, pp) and declined over time only in urban non-slum households. Among adults, underweight prevalence decreased by ~13 pp but overweight/obesity doubled (10% to 21%) between 2006 and 2016. Rises in overweight/obesity among women were greater in rural and urban slum than urban non-slum households. Within-residence, wealth inequalities were large for both underweight (SII -35 to -12pp) and overweight/obesity (+16 to +29pp) for adults, with the former being more concentrated among poorer households and the latter among wealthier households. In conclusion, India experienced a rapid decline in child and adult undernutrition between 2006 and 2016 across genders and areas of residence. Of great concern, however, is the doubling of adult overweight/obesity in all areas during this period and the rise in wealth inequalities in both rural and urban slum households. With the second largest urban population globally, India needs to aggressively tackle the multiple burdens of malnutrition, especially among rural and urban slum households and develop actions to maintain trends in undernutrition reduction without exacerbating the rapidly rising problems of overweight/obesity.

## Introduction

Urban expansion is occurring rapidly in many parts of the world. Whereas approximately 55% of the world’s population currently lives in urban areas, this figure is expected to rise to 60% in 2030 and 68% by 2050 [1]. Urban populations often grow faster than the capacity of cities to support them, with slum populations increasing as a result, particularly in South Asia. An estimated 881 million people worldwide lived in slums in 2014, and this number is projected to increase to two billion in 2030 [2]. India has the second largest urban population in the world; the proportion of India’s population living in urban areas is expected increased from 34% (460 million) in 2018 to 40% (607 million) in 2030 and 53% (876 million) in 2050 [1]. Several large cities in India currently have more than 40% of their population residing in slums [3].

As the world’s population becomes more urbanized, many challenges related to the rapid expansion of urban areas and the explosion of slums arise, including overcrowding, lack of basic services, substandard housing, limited access to healthcare, unsafe water, and inadequate sanitation [4]. Among the many health and nutrition challenges faced by urban dwellers, the double burden of malnutrition–i.e. the coexistence of undernutrition and micronutrient deficiencies and of overweight/obesity–is becoming a major public health concern globally and in urban areas in particular [5, 6]. Urbanization is a strong driver of the double burden of malnutrition because it causes remarkable shifts in urban food systems (particularly the availability of cheap ultra-processed food and beverages), in urban diets (convenient and processed foods), and in lifestyles (lower levels of physical activity related to the labor-saving technologies, more sedentary work, more leisure, and motorized transportation) [5].

While many studies from low- and middle- income countries (LMICs) have reported malnutrition disparities between socioeconomic groups or by urban–rural residence, very few have examined the intersection between wealth and area of residence, and none have done so in India (S1 Table in S1 File). Understanding inequalities in different forms of malnutrition related to wealth status and residence is vital to identifying solutions to achieving equality, a core cross-cutting theme of the Sustainable Development Goals.

In India, health has improved in recent decades, but progress has been uneven and inequitable. Maternal and child undernutrition disproportionately burdens the poor [7, 8], especially the urban poor where undernutrition and other health conditions are worse than among their wealthier counterparts [9, 10], and sometimes even worse than in rural areas [11]. Urban slum-dwellers are among the most vulnerable population groups and face high mortality and undernutrition, as well as limited access to maternal and child health care services [11, 12]. Furthermore, slum-dwellers are burdened by the low-quality of public service provision such as access to water, sanitation, decent housing, and health insurance [13–15]. Despite these widening gaps, understanding inequalities in the different forms of malnutrition in India has not received sufficient attention. Most health inequality studies used previous National Family Health Survey (before 2006) and some used data on some select states, but no study to our knowledge has examined trends and wealth inequalities within place of residence over time. Our study aimed to fill this gap by analyzing trends and inequalities in child and adult undernutrition and overweight/obesity by gender, area of residence, and wealth in India from 2006 to 2016.

## Methods

### Data sources

We used data from two rounds of nationally representative surveys—the National Family Health Surveys in 2005–2006 (NFHS-3) [16] and 2015–2016 (NFHS-4) [17]. Both surveys follow a systematic, multi-stage stratified sampling design used in Demographic and Health Surveys in many other countries. The first stage involved selection of primary sampling units (i.e. villages in rural areas and Census Enumeration Blocks in urban areas) using probability proportional to population size. The second stage involved the random selection of 22 households from each primary sampling unit. These surveys gathered demographic, health and nutrition data on 109,041 and 601,509 sample households, respectively. While NFHS-3 is representative at the state level, NHFS-4 is representative at both state and district levels, and both surveys are also representative at urban/rural levels. In this study, we used data from girls 0–59 mo (n = 24,756 for 2006, 124,525 for 2016), boys 0–59 mo (n = 26,799 for 2006, 135,102 for 2016), women 15–49 y (n = 118,474 for 2006, 667,258 for 2016) and men 15-54y (n = 74,338 for 2006, 112,122 for 2016) with available anthropometric measurements.

### Outcomes

The outcomes of interest were anthropometric measures for girls, boys, women and men. Children’s weight and length/height measurements were used to derive z-scores by comparing each child’s anthropometric measurements to the WHO age- and gender-appropriate child growth standards. Two indicators were calculated: length/height-for-age z-score (HAZ) and body mass index-for-age z-score (BMIZ, using children’s weight and height for age). Stunting was defined as HAZ < -2 and overweight as BMIZ >1. The height and weight measures of women and men were used to calculate body mass index (BMI) in kg/m^2^. Underweight was defined as BMI <18.5 kg/m^2^ and overweight as BMI ≥25 kg/m^2^. We use a combination of overweight and obesity given extensive epidemiological research highlighting the risks of noncommunicable diseases associated with BMI ≥25 kg/m^2^ [18].

### Definition of rural, urban slum and urban non-slum households

We compared outcomes in rural, urban slum and urban non-slum households. The urban and rural clusters in NFHS were chosen using the classifications of the Census of India 2011 [19]. Urban areas were defined as Statutory Towns (including all places with a municipality, corporation, cantonment board or notified town area committee) and Census Town (including all other places with a minimum population of 5,000; at least 75% of the male working population engaged in non-agricultural pursuits; and a density of population of at least 400 persons/square kilometer).

There is no agreement on how to define and hence identify a slum (S2 Table in S1 File). After reviewing literature on slum definitions, we applied the UN-HABITAT definition [20, 21] which ascribes a household to a slum based on satisfying at least two of the following four criteria: 1) overcrowding/insufficient living space (three or more people sharing a room), 2) non-durable housing (if the wall, roof or floor of the house is built with unimproved materials), 3) lack of access to improved and adequate safe drinking water, and 4) lack of access to adequate sanitation facility. All these criteria applied for households defined as urban in NFHS only. We compared the percentage of slum households using our definition to the percentage of identified slums reported in NFHS-3 and NFHS-4 in 8 large cities and found similar results.

### Wealth index

A wealth index was constructed using pooled data from 2006 and 2016 to compare wealth over time. A principal component analysis was used to construct the index, which included household ownership of 17 assets (car, motorbike, bicycle, television, radio, computer, refrigerator, mobile phone, watch, fan, bed, mattress, table, chair, pressure cooker, sewing machine, water pump), livestock (cow, goat, chicken), house and land [22]. The first component derived from the component scores explained ~80% of the variance and was then used to categorize wealth into quintiles for urban slum, non-slum and rural separately; the lowest quintile (Q1) represents the poorest 20% and the highest quintile (Q5) represents the richest 20% of the pooled population.

### Data analysis

Descriptive analyses were used to summarize sample characteristics. Graphical methods were used to visualize how stunting in children, underweight in adults, and overweight in both children and adults differed by residential areas over time. These changes were examined using absolute change, the percentage point (pp) difference between the two surveys. The statistical significance of changes between 2006 and 2016 was tested using an adjusted Wald test.

To examine inequalities in the burden of malnutrition by wealth index within residential areas, we first used equity plots disaggregated by residence and wealth quintile for each age/sex group, to visualize the prevalence of outcomes in 2006 and 2016. We then examined absolute and relative wealth inequalities for each outcome by residential area using the slope index of inequality (SII) and the concentration index (CIX) [23, 24]. These two complex measures are widely used to summarize health inequality in a series of subgroups such as wealth quintiles, taking into account the entire distribution of outcomes over the five quintiles, and weighted by the sample size of each quintile. SII represents the absolute difference (percentage points, pp) in predicted values of outcomes between the two extremes of the wealth distribution (lowest and highest wealth quintile). CIX is a relative measure of inequality; it is related to the Gini coefficient and can be expressed in the form of a curve that ranks the sample wealth index on the x-axis and plots cumulative health outcomes on the y-axis. Both SII and CIX take values from -100 to +100, where positive values indicate the outcome is more prevalent among the rich and negative values indicate the outcome is more prevalent among the poor. These two inequality measures were also used to assess changes in inequalities over time. All analyses were performed using Stata version 15.1. All models were adjusted for age, cluster sampling design and survey sampling weights. Two-sided P<0.05 was used for statistical significance.

### Ethics statement

This study was a secondary data analysis of NFHS data, which was approved by the institutional review board of the International Institute of Population Sciences, India. All respondents in the NFHS undergo an informed consent process for participation in the survey. The enumerators read a detailed informed consent statement to the respondents, informing about the survey, describing the procedure, and emphasizing the voluntary nature of participation. Once the respondent agreed to participate in the survey, the interviewer signed the questionnaire to indicate that the informed consent statement had been read to the respondent.

## Results

### Sample characteristics

In 2006, two thirds of households lived in rural, 26% in non-slum and 7% in slum areas (Table 1). As per survey design, the share of households in each residential area was similar between 2006 and 2016. Most houses in rural clusters were built of non-durable material (71% in 2006 and 57% in 2016), were crowded (42% in 2006 and 35% in 2016) and lacked an adequate sanitation facility (77% in 2006 and 57% in 2016). The prevalence of insufficient living space was highest for urban slum dwellers (~72% in both years). Wealth and living conditions improved over time for all three residential areas.

**Table 1:** Characteristics of households, girls, boys, women and men by survey round and residential area, India 2006 and 2016.

	Overall		By residential area					
	2006	2016	2006			2016		
			Rural	Urban Slum	Urban Non-Slum	Rural	Urban Slum	Urban Non-Slum
**Household level**								
Number of households	109,041	601,509	58,805	9,982	40,254	425,563	33,312	142,634
Share of households (%)	100.0	100.0	67.4	6.8	25.8	65.1	5.7	29.2
Non-durable housing^1^ (%)	53.9	41.8***	71.1 ^a^	67.8^b^	5.5^c^	57.1^b^	56.6^b^	6.0^a^
Lack of access to adequate sanitation facility^2^ (%)	58.4	41.7***	76.5 ^b^	73.8 ^b^	7.1^a^	56.7^b^	57.5^b^	4.8^a^
Lack of access to safe drinking water^3^ (%)	12.4	10.6***	15.6^b^	15.2^b^	3.3^a^	11.2^c^	26.9^b^	6.2^a^
Insufficient living space (%)	40.3	32.3***	42.2^c^	71.8 ^b^	26.9^a^	34.6^c^	71.5^b^	19.4^a^
Wealth index	-0.5	0.05***	-0.88^c^	-0.63^b^	0.51^a^	-0.27^c^	-0.05 ^b^	0.80^a^
**Girls 0–5 y**								
N	24,756	124,525	15,504	3,023	6,229	95,276	8,471	20,778
Age, mo	29.5	29.7	29.3 ^b^	29.6^a^^,^ ^b^	30.1^a^	29.6 ^a^	29.9^a^	30.0^a^
**Boys 0–5 y**								
N	26,799	135,102	16,568	3,182	7,049	102,972	9,058	23,072
Age, mo	29.7	29.8	29.7 ^a^	30.5 ^a^	29.6^a^	29.5^b^	30.4^a^	30.4^a^
**Women 15–49 y**								
N	118,474	667,258	63,786	11,561	43,127	470,341	40,829	156,088
Age, y	29.3	30.2***	29.2 ^c^	28.8^b^	30.0 ^a^	30.0 ^c^	29.5 ^b^	31.0 ^a^
**Men 15–54 y**								
N	74,338	112,122	36,155	8,337	29,846	76,596	6,993	28,533
Age, y	31.1	31.9***	31.1 ^a^	30.8 ^a^	31.0^a^	31.7^c^	31.1 ^b^	32.3 ^a^

1 A house is categorized as non-durable if the wall or the roof or the floor of the house is built with unimproved materials

2Unimproved sanitation facilities include—flush or pour-flush to elsewhere, pit latrine without slab or open pit, bucket, hanging toilet or hanging latrine, no facilities, or bush or field

3Unimproved water sources include unimproved dug well, unprotected spring, cart with small tank/drum, bottled water, tanker-truck, surface water (river, dam, lake, pond, stream, canal, irrigation channels)

*** Significant difference between 2006 and 2016, *** *p* < 0.001

^a, b, c^Labeled values in a row without a common superscript letter differ among urban non-slum, urban slum and rural, *p* < 0.05.

### Differences in stunting, underweight, and overweight/obesity by residential area and time

**Fig 1:**
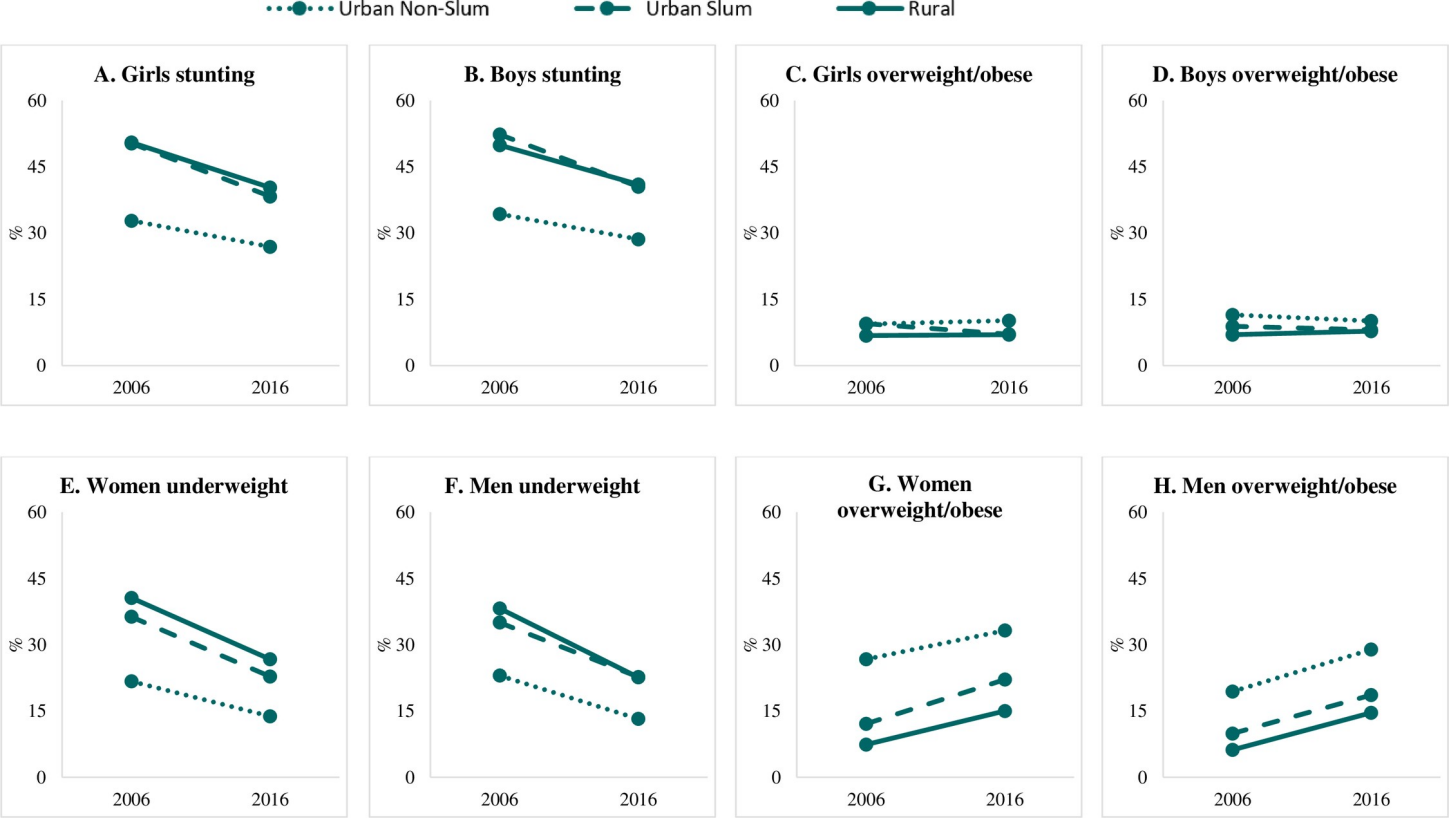
Prevalence and absolute change in undernutrition and overnutrition among Indians by residential area, 2006 to 2016. (A) Girls (0-5y) stunting, (B) Boys (0-5y) stunting, (C) Girls (0-5y) overweight/obese, (D) Boys (0-5y) overweight/obese, (E) Women (15-49y) underweight, (F) Men (15-54y) underweight, (G) Women (15-49y) overweight/obese, (H) Men (15-54y) overweight/obese.

Child stunting dropped by 10 percentage points (from 48% to 38%) over the 10-year period from 2006 to 2016 (Table 2), with no statistically significant differences between boys and girls. The reduction in stunting was significantly higher among children from rural and urban slum households (-9 to -12 pp, depending on area and sex) than among those in urban non-slum households (-6 pp) (Fig 1 and Table 2). At both time points, stunting was similarly high among children from rural and urban slum households (~50% in 2006 and ~40% in 2016), but much lower among those from urban non-slums (33% in 2006 and 27% in 2016 for girls, 34% in 2006 and 29% in 2016 for boys).

**Table 2:** Nutrition outcomes for children and adults by survey round and residential area, India 2006 and 2016.

Indicators	Area	Year		Change	
		2006	2016	Absolute change (2016–2006, pp)	Relative change ([2016–2006]/2006, %)
**Children 0–5 y**					
Girls stunting^1^	Rural	50.5^a^	40.3 ^a^	-10.2*** ^a^	-20.2
	Urban slum	50.3^a^	38.3 ^b^	-12.0*** ^a^	-23.9
	Urban non-slum	32.8^b^	26.9 ^c^	-5.9*** ^b^	-18.0
	All girls	47.6	37.5	-10.1***	-21.2
Boys stunting	Rural	49.9 ^a^	41.0 ^a^	-8.9*** ^a^	-17.8
	Urban slum	52.3 ^a^	40.5 ^a^	-11.8*** ^a^	-22.6
	Urban non-slum	34.3 ^b^	28.6 ^b^	-5.7*** ^b^	-16.6
	All boys	47.5	38.4	-9.1***	-19.2
Girls overweight/ obese^1^	Rural	6.8 ^a^	7.0 ^a^	0.2 ^a^	2.9
	Urban slum	9.5 ^a^	7.1 ^a^	-2.4* ^b^	-25.3
	Urban non-slum	9.4 ^b^	10.2 ^b^	0.8 ^a^	8.5
	All girls	7.4	7.7	0.3	4.1
Boys overweight/ obese^1^	Rural	7.0 ^a^	7.8 ^a^	0.8* ^a^	11.4
	Urban slum	8.9 ^a^^b^	8.1 ^a^	-0.8 ^a^^b^	-9.0
	Urban non-slum	11.5 ^b^	10.1 ^b^	-1.4 ^b^	-12.2
	All boys	7.9	8.3	0.4	5.1
**Adults 15–54 y**					
Women (15-49y) underweight^1^	Rural	40.6 ^a^	26.7 ^a^	-13.9*** ^a^	-34.2
	Urban slum	36.3 ^b^	22.8 ^b^	-13.5*** ^a^	-37.2
	Urban non-slum	21.7 ^c^	13.8 ^c^	-7.9*** ^b^	-36.4
	All women	35.6	22.9	-12.7***	-35.7
Men (15-54y) underweight^1^	Rural	38.2 ^a^	22.6 ^a^	-15.6*** ^a^	-40.8
	Urban slum	35.0 ^b^	22.7 ^a^	-12.3*** ^b^	-35.1
	Urban non-slum	23.0 ^c^	13.2 ^b^	-9.8*** ^c^	-42.6
	All men	33.8	19.8	-14.0***	-41.4
Women (15-49y) overweight/ obese^1^	Rural	7.4 ^a^	15.0 ^a^	7.6*** ^a^	102.7
	Urban slum	12.1 ^b^	22.1 ^b^	10.0*** ^b^	82.6
	Urban non-slum	26.7 ^c^	33.2 ^c^	6.5*** ^c^	24.3
	All women	12.5	20.5	8.0***	64.0
Men (15-54y) overweight/ obese^1^	Rural	6.2 ^a^	14.6 ^a^	8.4*** ^a^	135.5
	Urban slum	9.9 ^b^	18.6 ^b^	8.7*** ^a^	87.9
	Urban non-slum	19.4 ^c^	28.9 ^c^	9.5*** ^a^	49.0
	All men	10.1	19.1	9.0***	89.1

*** Significant difference between 2006 and 2016

*P<0.*5*, *** *P* < 0.001

^a, b, c^Labeled values in a column without a common superscript letter differ among urban non-slum, urban slum and rural within year, *P* < 0.05. All models controlled for age, gender, cluster sampling design and survey sampling weights.

Overweight/obesity affected ~8% of children and did not change over time. Overweight/obesity prevalence was slightly lower (by ~3–4 pp) in rural compared to slum and non-slum households for both girls and boys.

The prevalence of underweight also declined markedly between 2006 and 2016, from 36% to 23% among women and 34% to 20% among men (Fig 1 and Table 2). Larger declines were observed among adults living in rural (14–16 pp), followed by urban slum (12–14 pp) and non-slum dwellers (8–10 pp). By contrast, the prevalence of overweight/obesity in adults almost doubled between 2006 and 2016 (from 10 to 21%) and the relative increases were higher in adults from rural households (103–136%) compared to those from slum (83–88%) and non-slum households (24–49%) (Table 2).

### Wealth inequality in stunting, underweight and overweight/obesity by gender and residential area

**Fig 2:**
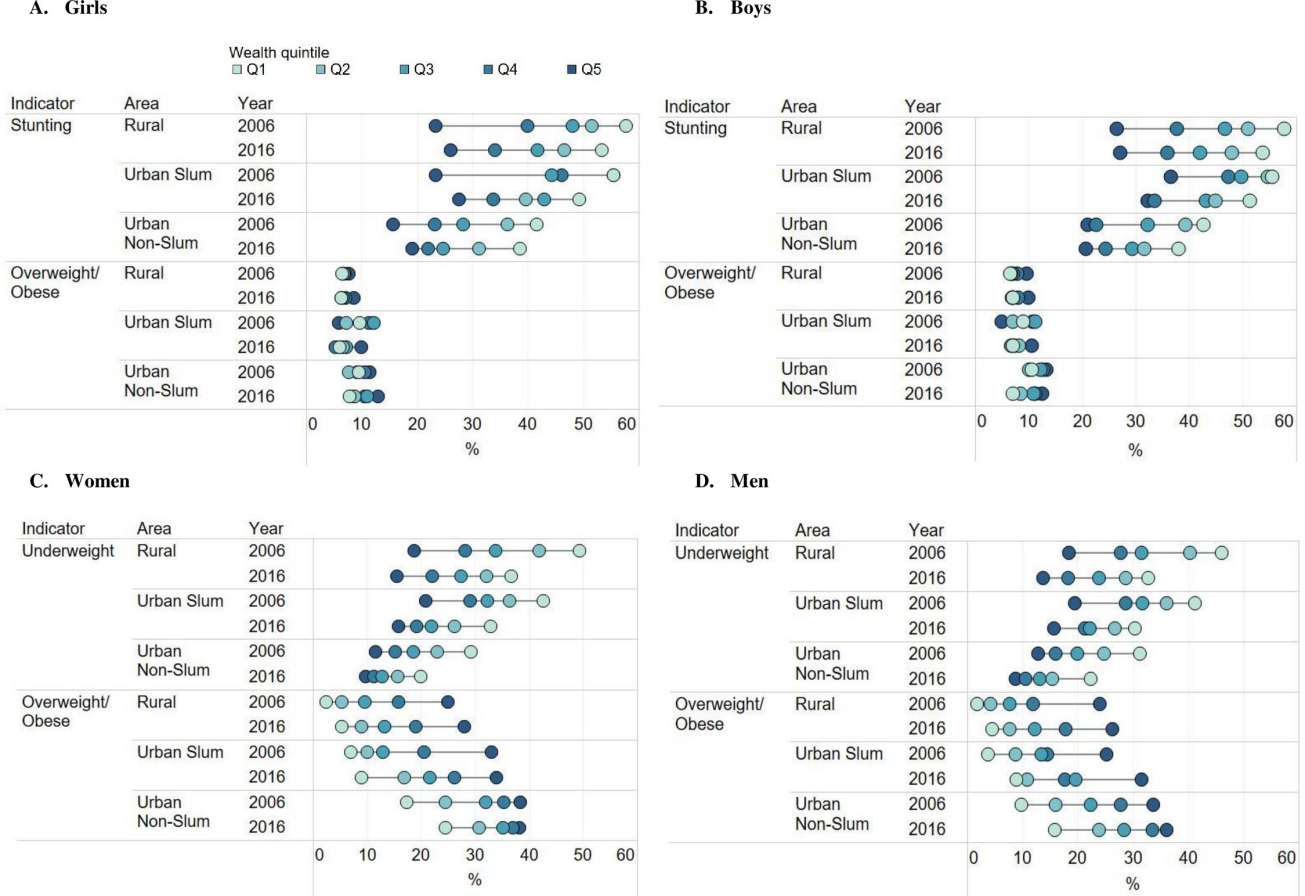
Socio-economic and residential inequality^1^ in undernutrition and overnutrition among Indian children and adults, 2006 and 2016. (A) Girls (0-5y), (B) Boys (0-5y), (C) Women (15-49y), (D) Men (15-54y). ^1^Within each residential area in a given year, each dot represents the prevalence of a given burden for a quintile subgroup and the spread of the dots indicates the inequality in the burden of malnutrition; ^2^Negative values mean that the burden is more concentrated in the poor and positive values mean that the burden is more concentrated in the wealthy.

Child stunting prevalence was higher among poorer compared to wealthier households across all residential areas and survey years, as shown by the equity plots and negative SII and CIX (Fig 2 and Table 3). Large wealth gaps (Q5 vs. Q1) were found among girls and boys in all residential areas and in both years (SII ranged from -33 to -19 pp). The inequality gaps in stunting decreased between 2006 and 2016 in urban non-slum households but did not statistically significant change over time for rural or urban slum households. Regarding overweight/ obese, wealth gaps were small for children in both rural or urban slum households (SII: 0.8 to 7pp) but increased slightly in urban non-slum households between 2006 and 2016.

**Table 3:** Inequality gaps in stunting, underweight and overweight among children and adults in India from 2006 and 2016, by wealth quintile and residential area.

	Area	Year	Q1	Q5	SII		CIX	
Children 0–5 years			%	%	SII	p^a^	CIX	p^a^
Girls stunting^1^	Rural	2006	57.7	23.3	-30.7***	0.32	-19.2***	0.16
		2016	53.3	26.1	-32.8***		-21.2***	
	Urban slum	2006	55.4	23.4	-24.2***	0.96	-18.2***	0.58
		2016	49.3	27.6	-24.5***		-16.0***	
	Urban non-slum	2006	41.6	15.6	-30.3***	0.10	-19.6***	0.13
		2016	38.7	19.1	-23.2***		-15.3***	
Boys stunting	Rural	2006	57.8	26.5	-32.2***	0.98	-19.8***	0.36
		2016	53.8	27.0	-32.3***		-21.1***	
	Urban slum	2006	55.6	36.6	-19.2**	0.18	-10.7**	0.27
		2016	51.3	32.2	-23.9***		-15.6***	
	Urban non-slum	2006	42.6	21.1	-28.2***	0.02	-19.7***	0.007
		2016	38.0	20.8	-19.9***		-13.4***	
Girls overweight/ obese^1^	Rural	2006	6.5	7.7	0.8	0.12	0.5	0.12
		2016	6.4	8.6	2.7***		1.7***	
	Urban slum	2006	9.6	5.9	6.0	0.45	1.0	0.68
		2016	6.1	9.9	3.4		2.0	
	Urban non-slum	2006	9.5	11.4	1.9	0.22	1.8	0.24
		2016	7.9	13.0	5.8***		4.3***	
Boys overweight/ obese^1^	Rural	2006	6.5	9.6	2.6*	0.40	0.8	0.05
		2016	7.0	9.9	3.6		2.3***	
	Urban slum	2006	9.0	5.0	6.5	0.44	0.1	0.40
		2016	12.1	7.4	3.3		2.0	
	Urban non-slum	2006	10.5	13.3	3.6	0.24	2.7*	0.31
		2016	7.0	12.5	6.7***		4.4***	
**Adults 15–54 years**								
Women (15-49y) underweight^1^	Rural	2006	49.3	18.7	-34.8***	<0.001	-19.5***	<0.001
		2016	36.7	15.5	-25.7***		-16.9***	
	Urban slum	2006	42.7	20.8	-22.3***	0.42	-15.1***	0.29
		2016	32.8	15.9	-19.6***		-12.8***	
	Urban non-slum	2006	29.2	11.5	-22.0***	<0.001	-14.9***	<0.001
		2016	20.0	9.8	-11.9***		-8.1***	
Men (15-54y) underweight^1^	Rural	2006	46.2	18.5	-30.9***	<0.001	-19.0***	<0.001
		2016	32.7	13.8	-23.2***		-14.9***	
	Urban slum	2006	41.3	19.6	-22.5***	0.19	-15.5***	0.10
		2016	30.3	15.8	-16.6***		-10.8***	
	Urban non-slum	2006	31.3	12.9	-23.1***	<0.001	-15.9***	<0.001
		2016	22.4	8.8	-14.4***		-9.2***	
Women (15-49y) overweight/ obese^1^	Rural	2006	2.4	24.9	23.5***	<0.001	13.3***	<0.001
		2016	5.3	28.1	28.4***		17.7***	
	Urban slum	2006	7.1	33.1	21.8***	<0.001	14.7***	0.02
		2016	9.0	33.9	29.3***		19.0***	
	Urban non-slum	2006	17.3	38.3	27.3***	<0.001	19.2***	<0.001
		2016	24.6	38.2	16.1***		10.5***	
Men (15-54y) overweight/ obese^1^	Rural	2006	1.9	24.1	20.7***	<0.001	11.9***	<0.001
		2016	4.6	26.3	27.8***		16.8***	
	Urban slum	2006	3.8	25.3	22.5***	0.26	14.9***	0.64
		2016	8.9	31.6	26.6***		15.9***	
	Urban non-slum	2006	9.8	33.7	28.6***	0.01	19.5***	<0.001
		2016	15.8	36.2	23.2***		14.5***	

*,**,***: significant difference for inequality between Q1 and Q5, *** p<0.001, ** p<0.01, * p<0.5 Q: quintile. SII: Slope Index of Inequality, CIX: Concentration Index

p^a^: p values for difference between 2006 and 2016

Adult underweight affects the poor more than the rich across all residential areas and survey years, as shown by the equity plots and negative SII and CIX (Fig 2 and Table 3). The wealth gaps in underweight among adults were large for all residential areas and in both years (SII -22 to -35 pp in 2006 and -12 to -23 pp in 2016). The wealth gaps in adult underweight were narrower in 2016 than in 2006 (p< 0.05) for both women and men in rural and non-slum households, mainly due to improvements among the poor in these residential areas.

Both women and men across all wealth quintiles and residential areas are becoming more overweight/obese over time, with the only exception of the highest quintile in urban non slums who have stabilized at an alarmingly high prevalence of about 40% (Fig 2). The CIX values for adult overweight/ obesity were all positive, indicating that the problem was more concentrated in the high wealth quintile households. The differences between wealth groups were large in all residential areas (SII: +16 to +29 pp) and increased between 2006 and 2016 in rural (SII: +21 to +28 pp) and urban slum areas (SII: +22 to +29 pp) between 2006 and 2016 but decreased in non-slum areas for both women and men (SII: +29 to +16 pp) (Table 3).

## Discussion

Although India has made progress in reducing undernutrition in the past decade, wealth inequalities within residential areas and the rapid rise in adult overweight/obesity are major challenges. India still has a large and relatively impoverished rural population, but also a rapidly growing urban population, of which a large proportion lives in urban slum areas. We have shown that the prevalence of stunting among children and underweight among adults (and the wealth inequalities within area of residence) have declined over time but remain significantly higher in rural and urban slum compared to urban non-slum households. By contrast, overweight/obesity among both men and women doubled over time in rural and urban slum households, reaching 15% and 22%, respectively, and 33% in urban non slums. Overweight/obesity are highly concentrated in upper wealth quintiles across all three areas of residence. Overweight/ obesity in children, however, remained at a relatively low 8% between 2006 and 2016.

India’s progress in reducing stunting among children aged 0–59 months (48% to 38%) likely reflects improvements in income and socioeconomic status, parental education, access to health services and improvements in water and sanitation [25]. We now show that stunting is almost as prevalent in urban slums as in rural households and, relative to these two areas, lower in urban non-slum households. Between 2006 and 2016, wealth inequalities in stunting declined in urban non-slum households but persisted in rural and urban slum households. Our findings highlight the precarious health and nutrition conditions of children living in poor urban households and call for renewed efforts to reduce inequalities in access to social and health programs for both urban slum and rural households [11]. Strategies to improve health and nutrition for urban slum households should be tailored to the specific needs of these populations and should be mainstreamed in the implementation of the Sustainable Development Goals and the New Urban Agenda [4].

Among adult men and women, we found large reductions in underweight from 2006 to 2016, especially in rural and urban slum areas, but a doubling in overweight/obesity over the same period. This is consistent with the global trend observed in LMICs, where the prevalence of underweight has declined substantially in the past few decades whereas rates of overweight/ obesity have increased [6]. We found an 8 pp increase in prevalence of overweight/obesity among Indian women (from 12.5% to 20.5%), compared to 5.6 pp among adult women in LMICs (from 24.4% to 30%) or 4.8 pp among adult women worldwide (from 34.4% to 39.2%) [26]. In our study, the relative increase in overweight/obesity was greater in rural compared to urban slum or urban non-slum households, a finding consistent with results from a previous report for women in LMICs [27]. The highest prevalence of adult overweight/obesity in India is currently found in urban non-slum households (at 33% for women and 29% for men) but, if past trends continue, rural and urban slum dwellers will quickly catch up to their urban counterparts. These findings point to the emergence of a significant double-burden of undernutrition and overnutrition in both rural and urban slum areas.

We also found large but narrowing wealth inequalities over time in underweight prevalence in all three residential areas, with similar patterns across adult men and women. India is unusual, compared to other countries, in having a high prevalence of underweight among both men and women, and a similar pattern emerging for overweight/obesity in 2016 which is only slightly lower among men (e.g. 22% and 19% in women and men living in urban slums and 33% and 29% respectively among those living in urban non-slums). Typically, overweight prevalence is higher among women compared to men at early stages of the nutrition transition [28, 29]; for example, women’s overweight prevalence in 2016 exceeds that of men’s by 16pp in Africa (39% vs 23%) and by 13pp in LMICs (32% vs.19%) [26]. These differences are mainly due to different contextual factors that drive gender differences in food consumption, physical activity, sedentary lifestyles, and sociocultural preferences and beliefs [28, 29]. We do not find any hypothesis to explain why women are not more overweight/obese than men in India.

While improvements in income and SES are key economic drivers of reductions in underweight among adults, these improvements are also contributing to the rapid rises in overweight/obesity [8, 30, 31]. A recent study in India showed that improvements in socio-economic status between 2006 and 2016 explain 29% of the reduction in underweight and 46% of the increase in overweight/obesity [8]. As incomes rise, people consume more diverse foods, a positive change, but also increase their consumption of processed foods and meals consumed away from home, thereby increasing their intake of energy, saturated fats and oils, added sugars, and salt [6]. Urbanization and economic growth are also typically associated with reductions in physical activity at work, in daily commute, and in leisure [6], which exacerbate the effects of increased energy consumption on overweight/obesity and diet-related non-communicable diseases.

India has a robust policy framework to address malnutrition in all forms [32]. Several evidence-based nutrition interventions in the first 1000 days are delivered through a wide range of national programs, including the Integrated Child Development Services, National Health Mission, Mid-Day Meal Scheme, Targeted Public Distribution System, and National Food Security Mission, among others. The 2017 National Nutrition Strategy [33] and POSHAN Abhiyaan, India’s national nutrition mission launched in early 2018, provide an updated strategic framework for action to improve nutritional outcomes by holistically addressing the multiple determinants of malnutrition through cross-sectoral convergence and contextualized planning at each level of the implementation process. Many policies and programs are heavily focused on undernutrition and on the rural poor given the historically high rates of maternal and child undernutrition especially in rural areas and the generally low prevalence of overnutrition. For example, early expansion of the National Rural Health Mission created more opportunities in rural areas than in urban areas [34] and the ICDS platform has expanded more in rural than urban areas between 2006 and 2016 [35]. It is now time for India to focus on all forms of malnutrition and on tackling the unique nutrition and health problems and inequalities of urban populations, especially those living in urban slums. New strategies need to shift to a focus on all forms of malnutrition, including undernutrition and micronutrient deficiencies as well as overnutrition (overweight and obesity and diet-related non-communicable diseases). Such interventions–referred to as “double-duty actions” [36, 37] have the potential to simultaneously tackle multiple forms of malnutrition and also prevent unintended negative consequences on overweight and obesity that has been shown to arise from programs aimed at reducing food insecurity and undernutrition in countries undergoing a rapid nutrition transition [38].

One potential limitation of this study relates to the definition of slums. Because there is no widespread consensus on the definition of slums, we used a definition that bears similarities to many others used in the empirical literature on slums, but we cannot rule out the possibility that our results may be sensitive to the choice of definition. With our definition, we found that 5% of the sample was classified as living in urban slum areas, which is consistent with the government estimates of 5.4% [3]. However, there is still uncertainty surrounding the true population-level prevalence of slum dwellers, making it difficult to validate our definition. One advantage of the NHFS is that it over-sampled slum areas of eight major cities, but it may have under-sampled slums in other urban centers of India. Another limitation is that our child-level analysis is restricted to children under five years old, whereas overweight prevalence may emerge in later childhood.

Due to the widespread burden of malnutrition and its importance for public health, research in India has increasingly focused on this topic, but studies have typically examined the effects of residential area [11, 39, 40] or socioeconomic status [30, 41, 42] separately. Our study is novel in examining the evolution of the double burden of malnutrition across distinct areas of residence and wealth quintiles within area. We use two rounds of large nationally representative data sets to explore trends and inequalities for both undernutrition and overnutrition for both children and adults. NFHS has several strengths in this regard, including standard measures of anthropometry and consistent scope for SES measures through well-established asset indices [22]. For inequality analyses, we used complex measures (SII and CIX) that account for the overall frequency and change in the outcomes over time and include weighting by sample size.

## Conclusion

The profile of malnutrition in India is changing rapidly, with progress on several indicators of undernutrition but rapidly rising rates of overweight/obesity, especially among adults. In addition to this double burden of malnutrition, India is characterized by significant health inequalities across socioeconomic groups and areas of residence and has made very limited progress in redressing these inequalities. The persistence of undernutrition alongside rising overweight/obesity suggests that India needs to develop new nutrition strategies that prioritize double-duty actions [36]. Potential interventions to consider in the Indian context include measures to promote positive changes in diets and physical activity, interventions to stimulate the demand for nutritious foods complemented by agri-food system innovation to increase their affordability, and regulatory approaches to discourage production, marketing and consumption of unhealthy, ultra-processed foods, beverages, and snacks. Future research should consider the efficacy of double duty actions to address both undernutrition and overweight/obesity and contextualize these actions to India’s diverse socioeconomic and environmental circumstances.

## Supporting information

S1 File (DOCX)
