# Supplementary material for: The double burden of malnutrition in India: Trends and inequalities (2006–2016)
Source: PLoS One. 2021 Feb 25;16(2):e0247856. doi: 10.1371/journal.pone.0247856 (PMC7906302; doi:10.1371/journal.pone.0247856)
Supplement: S1 File — (DOCX) [file pone.0247856.s001.docx]

**S1 Table: Systematic literature review of evidence for association between wealth and residence and double burden of malnutrition^1^**

| **Author, year** | **Data** | **Objective** | **Results** |
| --- | --- | --- | --- |
| Jeyaseelan, 1997^1^ | Cross-sectional survey of children aged 5-7 years | The study investigates the impact of hygiene, housing and sociodemographic variables on acute malnutrition among children | - Risk of malnutrition was higher in rural areas compared to urban areas - Risk was higher for children living in thatched roof houses compared to pukka houses, family using dung or firewood fuel compared to LPG and for children |
| Chhabra, 2007^2^ | Cross-sectional survey of adults (>18 years) in Delhi | Distribution of BMI by gender, place of residence and wealth | - Overall 24.8% of the sampled were underweight, 19.4% overweight and 6.1% were obese. - Underweight was higher in rural areas and overweight was higher in urban areas |
| Ackerson, 2008^3^ | NFHS-2 | Prevalence of underweight and overweight among women in India by size of neighborhood (large city, small city, town and village) and neighborhood wealth | - Large difference is prevalence of women underweight between village (37.3pp) and large city (16.3pp) - Higher overweight prevalence in large city (30pp) compared to village (6.6pp) - Did not find the pattern of double burden malnutrition at the state or neighborhood level. |
| Hazarika, 2009^4^ | NFHS-3 | To provide a national representation of women’s reproductive health in the slum population in India | There are significant differences in the utilization pattern of reproductive health services among women in the slum and non-slum communities. Women in the slums were:   - less likely to use modern contraceptives, higher discontinuation rate - less completed 4ANC and less had ANC in the first trimester - less access to institutional delivery and skilled attendants - depended on the government facilities for reproductive health services |
| Garg, 2010^5^ | NFHS-2 and NFHS-3 | Prevalence of obesity among Indian women | - Obesity had a positive trend between the two NFHS rounds (increase of 24.5%) - Prevalence was highest for women between 40-49 years, residing in urban areas and having high qualification - The study suggested that although majority population of India is malnourished, obesity seems to be rising in urban areas |
| Guha-Khasnobis, 2010^6^ | NFHS-3 | Compare women’s status between rural and urban settings | - Urban women seem to be better empowered compared to their rural counterparts - Women workers in an urban economy can earn more income which has been related to decision-making in the family, these women might be less tied to traditional restrictions, such as food taboos during pregnancy - Urban women also have fewer children, and they and their children are much more likely to be literate than rural women and theirs - Services such as family planning may be more available in urban areas |
| Subramanyam, 2010^7^ | NFHS-1,2,3 | Prevalence and trend in undernutrition among children by wealth, maternal education, caste and place of residence | - The study did not find any evidence of association between undernutrition and place of residence after controlling for household wealth, maternal education and caste. |
| Misra, 2011^8^ | Cross-sectional survey of urban school going children (8-18 years) in 5 Indian cities | Examined obesity and abdominal obesity by measuring height, weight and waist circumference and comparison by gender, wealth and size of city population | - Based on WHO cut-offs, 18% children were found to be overweight and 5.3% were found to be obese and 4.5% were abdominal obese - Prevalence was higher for females and high economic status and residence in cities with population greater than 4 million were positively associated with overweight and obesity |
| Swaminathan, 2012^9^ | NFHS-2 | Examined the association between slum residence and BMI among women in India. Compared the results between competing definitions of census and NFHS slums. | - Based on census definition of slum, there was no relation between slum residence and BMI while the opposite was true when the NFHS definition of slums was used - The study also found positive association between higher education, better economic status and risk of being overweight |
| Prakash, 2013^10^ | NFHS-3 | Compares the utilization of selected maternal and child health care services between the urban poor and non-poor in India | - Indicators of maternal and child health care are worse among the urban poor than in their non-poor counterparts: the levels of antenatal care, safe delivery and childhood vaccinations are much lower among the urban poor than non-poor, especially in socioeconomically disadvantageous states - The poverty status of the household emerged as a significant barrier to utilization of health care services in urban India |
| Gaur, 2013^11^ | NFHS-3 | Examines women’s nutritional status across eight mega cities with a specific focus on slum-non-slum divide | - The study found 50% of women in mega-cities are malnourished (either undernourished or overnourished) - Overweight was higher among non-slum residents and underweight was higher among slum dwellers - Regression results show no significant relation between place of residence and women’s nutritional status |
| Samal, 2015^12^ | Cross sectional survey of older Indians in 6 states | Estimate the prevalence of overweight and obesity among people above 50 years and compare results by residence, wealth, gender and occupation | - Study showed 14% older were overweight while 34% have central adiposity - Prevalence was higher in women, highly educated and urban areas - Study also showed that 25 percent in the poorest quintiles also had central adiposity |
| Sengupta, 2015^13^ | NFHS-2 and NFHS-3 | Prevalence and trend of overweight and obesity among every married women (15-49 years) from selected “underweight states” and “overweight states” in India by place of residence and wealth | - In overweight states, there is no rural-urban or rich-poor difference in proportion of women, although obesity is still higher for urban women (22% compared to 15%) - In underweight states, overweight and obesity is still higher in urban areas - The rate of rise of overweight and obesity is higher in rural areas of the “overweight states” |
| Usmani, 2018^14^ | Census reports, NFHS -3 | Examine the disparities within the urban population (slum and non-slum population) in health-related indicators | - Almost all of the urban poor population has less access to healthcare facilities as immunization, antenatal care, delivery by health professionals etc. - Infant and child undernutrition is also very high among the urban poor population or rather more than the rural population |
| Pörtner, 2018^15^ | NFHS -3 | Examine the association between area of residence (rural, urban and slum) and child health in India | - Rural Indian children have lowest HAZ, followed by slum and urban. - When controlled for wealth and health environment, slum children fare significantly worse than their rural counterparts - slum conditions (such as overcrowding and open sewers) are associated with 20% to 37% of slum children’s stunting risk |
| Dwarkanath, 2018^16^ | Prospective observational cohort, hospital-based study in Karnataka State | Investigate factors associated with BMI in early pregnancy of urban and rural South Indian women. | - Rural women were younger, had lower body weight, tended to be shorter and less educated - They lived in poor housing conditions, had less access to piped water and good sanitation, used unrefined fuel for cooking and had lower standard of living score |
| Luhar, 2018^17^ | NFHS 2, 3, 4 | Examine trends in prevalence of  overweight/obesity among adults in India by socioeconomic  position (SEP) between 1998 and 2016 | - Between 1998-2016, overweight/obesity prevalence increased among men and women in both urban and rural areas - In all periods, overweight/obesity prevalence was consistently highest among higher SEP individuals - In urban areas, overweight/obesity prevalence increased considerably over the study period among lower SEP adults |
| Luhar, 2019^18^ | NFHS 2, 3, 4 | Examined the socioeconomic (SES) patterning of overweight among adults in India’s most and least economically developed states between 1998 and 2016 | - There is an increasing trend of overweight prevalence among low SES women. - Amongst high SES women, overweight prevalence either increased to a smaller extent, remained the same or even declined between 1998 and 2016 |
| Vennu, 2019^19^ | NFHS4 | Calculate overweight, obesity, hypertension, and diabetes at national level and by age group, sex, and type of residence for each state and union territory | - The national prevalence of overweight, obesity, hypertension, and diabetes were 14.6%, 3.4%, 5.2%, and 7.1%, respectively. - The highest prevalence of these conditions/diseases was seen among those aged 35-49 years (54 years for men), especially women living in urban areas |
| Corsi, 2019^20^ | NFHS-4 | To conduct a comprehensive equity analysis of the SES gradients and distribution of diabetes, hypertension, and obesity in India using the latest national data. | - The magnitude of the SES gradient was strongest for obesity, followed by diabetes and hypertension - Analyses of the socioeconomic distribution indicated that between 70% and 90% of the population burden of diabetes, hypertension, and obesity was among the higher SES groups, and this figure was similar across states |
| Dutta, 2019^21^ | NFHS-4 | Assessed the determinants of underweight and overweight/obesity in India among adult men and women aged 15-49 | - Persistently high prevalence of underweight coexisting with an increased prevalence of over­weight/obesity in India - The risk of underweight was highest in the central and western regions. More educated and wealthier adults were less likely to be underweight - Overweight/obesity was more prevalent in urban areas, in the southern region, and among adults aged 35-49. Level of education and wealth index were positively associated with overweight/obesity. |
| Schott, 2019^22^ | Young lives study data from two cohorts: age one year to mid-adolescence and from mid-childhood to early adulthood | Examined and modelled trajectories of stunting and overweight in two age cohorts. Analyzed the heterogeneity in trajectories by child sex, place of residence, wealth index and mother’s education. | - Prevalence of stunting is relatively higher and persistent for the older cohort; it is declining for the younger cohort. Prevalence of overweight begins to increase by young adulthood in India. - Children that start with high stunting probability then recovers in adolescence. Overweight does not affect large proportion of children, but a few are embarking on its path during adolescence - Risk of child being in high stunting probability trajectory is lower for mothers with completed schooling, in top two wealth quintiles and urban residence. Overweight risk is higher in urban areas, top wealth quintile and being female |
| George, 2019^23^ | Cross-sectional survey of sample population in large slum of Bangalore | Studied household demographics and health conditions including malnutrition at the individual level | - 40% of the sampled population was obese and the majority of those were under 45 years. Females had higher obesity prevalence than males - Prevalence of anemia was 71.1% among adolescent girls - >30% of under 5-year children were underweight, >40% were stunted and >15% were wasted |
| Young, 2019^24^ | NFHS-3 and NFHS -4 | Examined the national and state trends for BMI and identified the determinants of underweight and overweight/obesity among adolescent girls and women | - Overall prevalence of underweight has decreased and overweight has increased. - The study also found that adolescents living in urban settings were 15% less likely to be underweight |
| Sethi, 2020^25^ | NFHS-3 and NFHS -4 | Presented the spectrum of malnutrition and their determinants for urban women | - Among urban poor mothers, 12.8% were short (height < 145 cm); 20.6% were thin (BMI < 18.5 kg/m2); 57.4% had anemia and 21.1% were overweight/obese (BMI ≥ 25 kg/m2). - Between 2006-2016, thinness reduced by 17 percentage points (pp), but obesity increased by 12 pp. |

^1^The search was conducted in PubMed on July 10, 2020. Search terms included (India AND (inequalities OR inequities OR disparities) AND ("double burden" OR malnutrition OR undernutrition OR underweight OR stunting OR overnutrition OR overweight OR obese OR obesity) AND (residence OR "residential area")) from 1976 to 2020 with no language filter; the search yielded 165 items, which were screened for relevance. Among the relevant items, references were also examined for additional relevant studies.

1. Jeyaseelan L, Lakshman M. Risk factors for malnutrition in south Indian children. *J Biosoc Sci* 1997; **29**(1): 93-100.

2. Chhabra P, Chhabra SK. Distribution and determinants of body mass index of non-smoking adults in Delhi, India. *J Health Popul Nutr* 2007; **25**(3): 294-301.

3. Ackerson LK, Kawachi I, Barbeau EM, Subramanian SV. Geography of underweight and overweight among women in India: a multilevel analysis of 3204 neighborhoods in 26 states. *Econ Hum Biol* 2008; **6**(2): 264-80.

4. Hazarika I. Women's reproductive health in slum populations in India: evidence from NFHS-3. *J Urban Health* 2010; **87**(2): 264-77.

5. Garg C, Khan SA, Ansari SH, Garg M. Prevalence of obesity in Indian women. *Obes Rev* 2010; **11**(2): 105-8.

6. Guha-Khasnobis B, James KS. Urbanization and the South Asian Enigma. A Case Study of India. Working Paper No. 2010/37. United Nations University. 2010.

7. Subramanyam MA, Kawachi I, Berkman LF, Subramanian SV. Socioeconomic inequalities in childhood undernutrition in India: analyzing trends between 1992 and 2005. *PLoS One* 2010; **5**(6): e11392.

8. Misra A, Shah P, Goel K, et al. The high burden of obesity and abdominal obesity in urban Indian schoolchildren: a multicentric study of 38,296 children. *Ann Nutr Metab* 2011; **58**(3): 203-11.

9. Swaminathan H, Mukherji A. Slums and malnourishment: evidence from women in India. *Am J Public Health* 2012; **102**(7): 1329-35.

10. Prakash R, Kumar A. Urban poverty and utilization of maternal and child health care services in India. *J Biosoc Sci* 2013; **45**(4): 433-49.

11. Gaur K, Keshri K, Joe W. Does living in slums or non-slums influence women's nutritional status? Evidence from Indian mega-cities. *Soc Sci Med* 2013; **77**: 137-46.

12. Samal S, Panigrahi P, Dutta A. Social epidemiology of excess weight and central adiposity in older Indians: analysis of Study on global AGEing and adult health (SAGE). *BMJ Open* 2015; **5**(11): e008608.

13. Sengupta A, Angeli F, Syamala TS, Dagnelie PC, van Schayck CP. Overweight and obesity prevalence among Indian women by place of residence and socio-economic status: Contrasting patterns from 'underweight states' and 'overweight states' of India. *Soc Sci Med* 2015; **138**: 161-9.

14. Usmani G, Ahmad N. Health status in India: A study of urban slum and non-slum population. *J Nurs Res Pract* 2018; **2**(1): 09-14.

15. Portner CC, Su YH. Differences in Child Health Across Rural, Urban, and Slum Areas: Evidence From India. *Demography* 2018; **55**(1): 223-47.

16. Dwarkanath P, Vasudevan A, Thomas T, et al. Socio-economic, environmental and nutritional characteristics of urban and rural South Indian women in early pregnancy: findings from the South Asian Birth Cohort (START). *Public Health Nutr* 2018; **21**(8): 1554-64.

17. Luhar S, Mallinson PAC, Clarke L, Kinra S. Trends in the socioeconomic patterning of overweight/obesity in India: a repeated cross-sectional study using nationally representative data. *BMJ Open* 2018; **8**(10): e023935.

18. Luhar S, Mallinson PAC, Clarke L, Kinra S. Do trends in the prevalence of overweight by socio-economic position differ between India's most and least economically developed states? *BMC Public Health* 2019; **19**(1): 783.

19. Vennu V, Abdulrahman TA, Bindawas SM. The Prevalence of Overweight, Obesity, Hypertension, and Diabetes in India: Analysis of the 2015-2016 National Family Health Survey. *Int J Environ Res Public Health* 2019; **16**(20).

20. Corsi DJ, Subramanian SV. Socioeconomic Gradients and Distribution of Diabetes, Hypertension, and Obesity in India. *JAMA Netw Open* 2019; **2**(4): e190411.

21. Dutta M, Selvamani Y, Singh P, Prashad L. The double burden of malnutrition among adults in India: evidence from the National Family Health Survey-4 (2015-16). *Epidemiol Health* 2019; **41**: e2019050.

22. Schott W, Aurino E, Penny ME, Behrman JR. The double burden of malnutrition among youth: Trajectories and inequalities in four emerging economies. *Econ Hum Biol* 2019; **34**: 80-91.

23. George CE, Norman G, Wadugodapitya A, et al. Health issues in a Bangalore slum: findings from a household survey using a mobile screening toolkit in Devarajeevanahalli. *BMC Public Health* 2019; **19**(1): 456.

24. Young MF, Nguyen P, Tran LM, Avula R, Menon P. A Double Edged Sword? Improvements in Economic Conditions over a Decade in India Led to Declines in Undernutrition as Well as Increases in Overweight among Adolescents and Women. *J Nutr* 2020; **150**(2): 364-72.

25. Sethi V, de Wagt A, Bhanot A, et al. Levels and determinants of malnutrition among India's urban poor women: An analysis of Demographic Health Surveys 2006 and 2016. *Matern Child Nutr* 2020; **16**(3): e12978.

**S2 Table: Literature review of slum definitions**

| **Title** | **Year of Publication** | **Authors** | **Definition of Slum** |
| --- | --- | --- | --- |
| The Challenge of Slums - Global Report on Human Settlements | 2003 | United Nations Human Settlement Program (UN-Habitat) | ‘Any specific place, whether a whole city, or a neighborhood, is a slum area if half or more of all households lack improved water, improved sanitation, sufficient living area, durable housing, secure tenure, or combinations thereof’  1. Inadequate access to safe water;  2. Inadequate access to sanitation and other infrastructure;  3. Poor structural quality of housing;  4. Overcrowding;  5. Insecure residential status. |
| Delhi's Slum Dwellers - Deprivation,  Preferences and  Political Engagement  among the Urban  Poor | 2012 | Banerjee, Abhijeet;  Pande, Rohini;  Walton, Micheal; | Nine criteria for Defining a Slum (at-least five should be marked for existence of a slum) 1. High Density of Housing 2. Poor Quality Housing Structure 3. Lack of Internal Housing Infrastructure 4. Poor Road Infrastructure 5. Access to water and water infrastructure 6. Uncovered and Unimproved Drains 7. Low coverage of private toilet facilities 8. High incidence of trash piles 9. Frequent cohabitation with animals |
| Primary Census Abstract for Slum | 2013 | Office of the Registrar General & Census Commissioner, India | - Areas notified by state, UT or local administration, housing and slum boards. - A compact area of at least 300 populations or about 60-70 households of poorly built congested tenements, in unhygienic environment usually with inadequate infrastructure and lacking in proper sanitary and drinking water facilities in the State/UT. |
| Urban Slums in India | 2014 | NSS 69th Round, Ministry of Statistics and Programme Implementation, Gov. of India | - Areas notified as slums by the concerned municipalities, corporations, local bodies or development authorities were termed notified slums. - Any compact settlement with a collection of poorly built tenements, mostly of temporary nature, crowded together, usually with inadequate sanitary and drinking water facilities in unhygienic conditions, was considered a slum by the survey, provided at least 20 households lived there. |
| Slum Residence and Child Health  in Developing Countries | 2014 | Günther Fink & Isabel Günther & Kenneth Hill | Households are slum like if lack two out of the four features:  1. Inadequate access to safe water;  2. Inadequate access to sanitation and other infrastructure;  3. Poor structural quality of housing;  4. Overcrowding;  Neighborhoods are categorized as slums if 75% of households lack two or more features |
| Slum Definitions in Urban India: Implications for the Measurement of Health Inequalities | 2015 | Laura B. Nolan | Household are categorized as ‘slum-like’ if they satisfy all 3 conditions: 1. Non-concrete roofing material 2. No drinking water facility on the premises 3. Use of public or no latrine  PSUs are defined as slums if over 50 percent households exhibited these characteristics |
| State of Slums in India, a statistical compendium | 2015 | National Buildings Organization | Any compact settlement with a collection of poorly built tenements, mostly of temporary nature, crowded together, usually with inadequate sanitary and drinking water facilities in unhygienic conditions, was considered a slum by the survey, provided at least 20 households lived there. |
| Understanding standard of living and correlates in  Slums: an analysis using monetary versus  Multidimensional approaches in three Indian cities | 2016 | Sugata Bag and Suman Seth | Criteria for defining a cluster as slum  1. Water facility: If the water source is non-improved 28 (UN-MDG) Or, stand-piped but time to fetch from source is 30 minutes or more Or, stand-piped but access duration is less than two hours per day 2. Sanitation facility: If there is no personal facility or the personal facility is shared with others 3. Type of house: If the wall or the roof or the floor of the house is built with unimproved materials; or there is no house 4. Leakage in house: If water enters in the house through roof or ground or both 5. Overcrowding: If more than three persons live per bedroom 6. Respiratory health risk: If biomass fuel is used or cooking is done inside sleeping room with no smoke outlet 7. Health insurance: If any member is suffering from chronic disease or there is any disabled member, And, no one in household has any health insurance scheme 8. Savings instrument: If no member in household has any instrument for savings 9. Asset ownership: If the household does not have any of the assets: washing machine, refrigerator, air conditioning machine, computer, four wheelers, and additional rent generating property in city 10. Information instrument: If the household does not have a land-line phone, and the number of mobile phones is less than the number of adults (15 years or more) in a household 11. Education attainment: If no household member has 10 or more years of schooling |
| Handbook of Urban Statistics | 2019 | Ministry of Housing and Urban Affairs, Government of India | Slums are those residential areas where dwellings are in any respect unfit for human habitation by reasons of dilapidation, overcrowding, faulty arrangements and designs of such buildings, narrowness or faulty arrangement of streets, lack of ventilation, light, sanitation facilities or any combination of these factors which are detrimental to safety, health and morals. A slum is characterized by lack of durable housing, insufficient living area, lack of access to clean water, inadequate sanitation and insecure tenure. |
| Because space matters: conceptual  framework to help distinguish slum  from non-slum urban areas | 2019 | Lilford R, Kyobutungi C,  Ndugwa R, *et al*. | Features that have been suggested as those that might help in characterizing slums   1. Built Environment: Durability of construction materials; Layout of lanes and orientation of structures—degree of entropy; Density (people sleeping in same room/people per square km) 2. Services: Water, Sanitation, Power, Solid waste management; health and education facilities. 3. Socioeconomic: Security of tenure title; Level of poverty; Crime and safety; Social Capital. 4. Ecology: Gradient; altitude (floodplains, areas at risk of subsidence, landslides and other hazards), Green spaces, Blue space, Air quality, Environment and industrial hazards |
